# Neglecting the fallow season can significantly underestimate annual methane emissions in Mediterranean rice fields

**DOI:** 10.1371/journal.pone.0198081

**Published:** 2018-05-31

**Authors:** Maite Martínez-Eixarch, Carles Alcaraz, Marc Viñas, Joan Noguerol, Xavier Aranda, Francesc Xavier Prenafeta-Boldú, Jesús Antonio Saldaña-De la Vega, Maria del Mar Català, Carles Ibáñez

**Affiliations:** 1 IRTA Institute of Agrifood Research and Technology, Aquatic Ecosystems, Sant Carles de la Ràpita, Spain; 2 IRTA Institute of Agrifood Research and Technology, GIRO, Torre Marimon, Caldes de Montbui, Spain; 3 IRTA Institute of Agrifood Research and Technology, Fruticulture, Torre Marimon, Caldes de Montbui, Spain; 4 IRTA Institute of Agrifood Research and Technology, Extensive crops, Amposta, Spain; RMIT University, AUSTRALIA

## Abstract

Paddy rice fields are one of the most important sources of anthropogenic methane. Improving the accuracy in the CH_4_ budget is fundamental to identify strategies to mitigate climate change. Such improvement requires a mechanistic understanding of the complex interactions between environmental and agronomic factors determining CH_4_ emissions, and also the characterization of the annual temporal CH_4_ emissions pattern in the whole crop cycle. Hence, both the growing and fallow seasons must be included. However, most of the previous research has been based on single-factor analyses that are focused on the growing season. In order to fill this gap, a study was conducted in a Mediterranean rice agrosystem (Ebre Delta, Catalonia) following a farm-to-farm approach with the purpose of 1) evaluating the cumulative and temporal pattern of CH_4_ emission, and 2) conducting a multi-variate analyses to assess the associative pattern, relative contribution and temporal variation of the main explanatory variables concerning the observed CH_4_ emissions. Measurements of CH_4_ emissions and agronomic and environmental parameters in 15 commercial rice fields were monitored monthly, during a whole crop field cycle. The temporal pattern of CH_4_ emission followed a bi-modal distribution peaking in August and October. The cumulative annual CH_4_ emissions from rice fields amounted 314 kg CH_4_ kg ha^-1^, of which *ca*. 70% were emitted during the fallow season. The main controlling factors of the CH_4_ emission rate in the growing season were positive related to water level and plant cover, while soil redox was negatively related. The main controlling factors in the fallow season were water level (negatively related, conversely to the growing season), as well as straw incorporation and soil temperature (positively related). The results of this study highlight the importance of the often neglected fallow season in the accurate estimation of CH_4_ emissions and, thus, the necessity of measurement programs that cover the whole crop field cycle. This information is the first step for setting effective mitigation strategies based on straw and water management.

## Introduction

Rice fields provide food for a half of the world population, particularly in developing countries subjected to intense demographic growth [1]. Methane (CH_4_) emissions from wetland rice cultivation contributes to a 9% of the anthropogenic emissions of this greenhouse-effect gas [2]. Hence, in order to meet the growing global food demand while reducing methane emissions, it is urgent to find mitigation strategies to minimize the impact of rice cultivation on climate change.

The determination of effective mitigation options in wetland rice cultivation must be based on a deep understanding of the processes involved in CH_4_ production and emission dynamics: from the underlying rhizosphere and plant physiology aspects to relevant environmental and agronomic factors that affect the temporal pattern of emissions. Improving the accuracy in the CH_4_ budget is fundamental to manage realistic and effective strategies to mitigate climate change. For this purpose, a more intense monitoring of CH_4_ emission at local scale is needed to provide accurate inventories [2].

The reported emissions in temperate and Mediterranean rice growing areas are highly variable because of the inherent spatial heterogeneity: in the USA, from 16.5 ± 11.4 kg C-CH_4_ ha^-1^ in Arkansas to 337.64 kg C-CH_4_ ha^-1^ in California [3,4]; in Italy, from 6.3 kg CH_4_ ha^-1^ to 537.6 kg CH_4_ ha^-1^ [5,6]; in Portugal and Spain, ca. 95 kg C-CH_4_ ha^- 1^ [7,8]; in Japan, from 121.4 kg to 431 kg [9,10]. Although Spain is a European leading rice producer [11], its inventory of rice-CH_4_ emissions is based on a single site study in Southern Spain (Sevilla) [7], what may compromise the representativeness of these estimations.

CH_4_ emissions are determined by complex interactions between environmental and agronomic factors involving the rice plants, soil and atmosphere [12], thus, a multidimensional approach is needed to correlate dynamics of CH_4_ emissions and environmental factors.

Several factors have been reported as important drivers regulating CH_4_ emissions, such as soil and air temperature [13], soil redox potential [14], fertilization [15] water level [16] and rice straw management [17,18], often with conflicting results. For instance, N fertilization has been identified both as a promoter [19] and as an inhibitor of CH_4_ emission [20]. Additionally, the effect of a particular factor (e.g. temperature) can be modulated by other biotic or abiotic conditions (e.g. water layer depth) [21].

The characterization of the temporal pattern of CH_4_ emissions is also important in order to identify the timing of emission peaks and, consequently to improve the understanding of CH_4_ dynamics and the efficacy of mitigation strategies. Furthermore, while most studies are focused on the growing season, emission data during the fallow season is scarce [21–23]. In some studies, the contribution of fallow to the total CH_4_ emission reached up to 50%, which demonstrates the need for year-round CH_4_ measurements to properly estimate the annual CH_4_ emissions [24].

Most of the previous research on major drivers of CH_4_ emissions has been focused on unifactorial (one-to-one) analyses of one or several factors. Hence, the multifactorial relationship among several parameters has been largely disregarded. Furthermore, most of those studies were conducted on a single field, so that the inherent variability of rice fields within a particular rice growing area was neglected.

The main objectives of this study are to assess the cumulative CH_4_ emissions in rice fields in an important rice growing area in the Mediterranean, the Ebre Delta, and to provide a first insight into the dynamics of CH_4_ emissions over both the growing and fallow seasons. Special attention was given to the assessment of the temporal variation and to the main controlling factors. For this purpose, 15 commercial rice fields spread all over the Ebre Delta, covering most of the agricultural, geographical and environmental variability, were selected and monthly monitored for CH_4_ emissions, agronomic practices, and soil physicochemical traits during a whole growth cycle period. The present study comprises the first comprehensive measurement program of methane emissions in the Ebre Delta and is expected to improve the understanding of the dynamics of CH_4_ emission in rice fields cultivated under Mediterranean conditions. The obtained results will contribute to improve the accuracy of the national and European inventories, and to the identification of key actions for the mitigation of CH_4_ emissions.

## Material and methods

### Study area

The Ebre Delta (Catalonia, NE Spain), with a total extension of 32.500 ha, is the third rice producing region in Spain, with 65% of its area covered by rice fields (21.125 ha). This activity is crucial for the economic sustainability economic activity of the area and for preserving the biodiversity of the surrounding natural wetlands. The climate of the region is Mediterranean with a mean annual precipitation of about 500 mm, mostly distributed during spring and autumn. The mean annual air temperature is 18°C with mild winters (mean temperature in January 9°C) and hot summers (mean temperatures in July 24°C).

Rice in the Ebre Delta is grown from late April to September and left fallow during the rest of the year. Water management during the growing season consists of permanent flooding from sowing time (late April-early May) to two weeks before harvest (September), with water layer of approx. 5 to 15 cm deep. During the vegetative and early reproductive stages, short periods of drainage can take place as a requirement for the application of herbicides. After harvest, fields are re-inundated for the incorporation of rice straw into the soil, which is the common management practices for this residue in the area. Thereafter fields are either flooded (from October to December) or left to progressively drain, according to the farmers’ preferences.

From January to March all rice fields are left dry for soil labour operations (harrowing and fertilizer application) and re-flooded in Mid-April, at the beginning of the cultivation period. Standard mineral fertilization is applied with average N doses ranging from 170 to 200 kg N ha^-1^. The rice cultivars used in this study were either Gleva or JSendra, which are representative Japonica-type cultivars in the Ebre Delta with medium grain size and a growth cycle of *ca*. 120 days from sowing to maturity. The maximum plant height of these cultivars is about 0.7 m.

### Experimental layout

**Fig 1:**
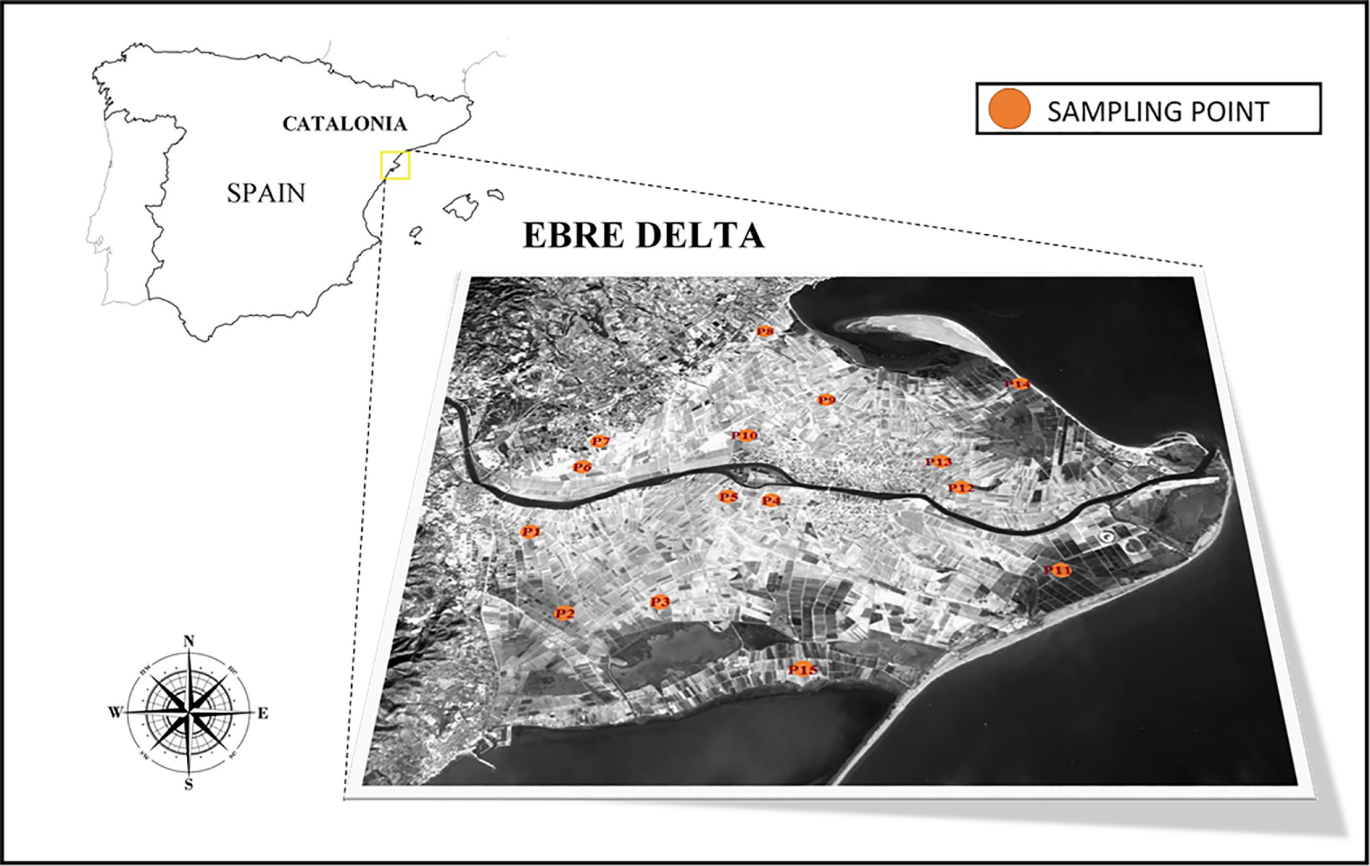
Map showing the study area, the Ebre Delta, and the location of the fifteen monitored commercial rice fields (P1 to P15). Landsat imagery courtesy of NASA Goddard Space Flight Center and U.S- Geological Survey.

Experiments were carried out in 2015, during the rice growing and post-harvest seasons (May to December), in 15 commercial rice fields spread over Ebre Delta (Fig 1). Authorization from landowners for sampling in their fields was obtained prior to the start of the sampling (S1 Text). These selected fields encompassed the environmental (soil properties and salinity, proximity to either river, sea or lagoons) and agronomic (water management, phytosanitary applications and agronomic performance) variability of the area. Periodical monitoring of CH_4_ emissions, soil physicochemical status and agronomic practices was carried out. The sampling size was set considering that 1) it should be larger than number of variables to conduct a multivariate analysis, 2) the time window for sampling was set to be within 10 am to 3 am to avoid diurnal variations as later described and 4) the practicability of sampling, i.e. manpower and costs.

The agricultural management of the 15 commercial fields followed the standard practices of the area as previously described.

### Gas sampling and flux measurement

Gas samples were monthly collected, from May to December, using the non-steady state chamber gas sampling method adapted from [25]. In summary, chambers consisted of polyvinylchloride (PVC) rectangular frames covered by transparent plastic and were dimensioned to fit the maximum height of the rice plants (12.9 m^2^ square base, 0.72 m height and 0.093 m^3^ of headspace volume. Chambers were equipped with two ports sealed with rubber septa, to avoid gas exchange during the insertion of a thermometer for temperature monitoring and a syringe during gas sampling. The air in the headspace was mixed by flushing with the syringe. Prior to the sampling, air in the headspace was mixed by flushing with the syringe. The basis of the chamber structure was covered by removable foam for its buoyancy on the flooded rice fields while preventing gas exchange between the headspace and the exterior. When the fields were dry, foams were removed and chambers were carefully placed on the soil, with humid towels around the base to prevent gas exchange. Blocks were installed in the field to support wooden boards to access the chamber while avoiding soil disturbance. These structures were installed one week before the first sampling and were placed at least at 1 m from the border of the field. Portable floating chambers were installed and removed every sampling day, ensuring the continuity of sampling in the event of damaged plants (chambers could be relocated over nearby healthy plants).

Every month, the 15 fields were sampled (S1 Table) within the same day and consistently from 10:00 am to 3:00 pm to minimize variability derived from the daily emission variation [26]. In this regard, every sampling day the order of field sampling was randomized. The field sampling order was randomized every sampling day. Three floating chambers [27] were installed per field and four gas samples (30 ml) per chamber were taken every 10 minutes over a 30-minute period. Each gas sample was transferred overpressured to pre-evacuated 12.5 mL vials (Labco Ltd., Buckinghamsire, UK) and sent to laboratory. CH_4_ concentration was determined using a THERMO TRACE 2000 (Thermo Fisher Scientific, USA) gas chromatograph equipped with a flame ionization detector (FID, Trace GC 2000, Thermo Finnigan, Germany).

The calibration of the gas chromatograph was carried out using a methane standard in nitrogen provided by Carburos Metalicos S.A. (Spain). Calibration standards have been prepared by successive dilutions in pure nitrogen, Carburos Metalicos S.A. (Spain), using a 1-liter Jumbo Syringe, SGE Analitical Science (Australia), and gas tight syringes of various sizes (from 500 μl to 5 ml), Hamilton (USA). The working range of the calibration curve has been adjusted depending on the methane concentration, from 3 to 30 ppm for the samples of low concentration, and from 30 to 600 ppm for the samples of high concentration.

Rates of CH_4_ emissions were calculated from the linear regression slope between CH_4_ concentration and the 30-minutes period of sampling in each chamber. CH_4_ concentration of each sample was corrected for the increase of temperature in the headspace of the chamber according to the ideal gas law since (the pressure was kept constant through a pipe connecting the headspace to the outside. Only significant linear regressions (*P* < 0.05 and R^2^>0.80) were accepted, and non-significant regressions were considered as zero emission rates. The obtained emission rate was averaged per field and month, and the cumulative emission rates (annual, growing and fallow season) were calculated by assuming constant emission rates over the entire month.

### Environmental and agronomic measurements

Simultaneously to gas sampling, agronomic (water flooding, water layer depth, plant cover and timing of straw incorporation) and environmental (temperature, soil pH, conductivity and redox potential) measurements, were done at *ca*. 10 cm depth by triplicate, next to each gas-sampling chamber (S1 Table). Hanna HI9126 for soil pH and redox and Spectrum Technologies Field Scout for soil conductivity.

In March, before flooding, three soil samples per field were taken randomly and pooled for soil characterization: texture and bulk density (by granulometry) and content of organic matter, organic carbon, sulphates, and nitrogen (by Spectromery).

### Statistical analysis

Principal component analysis (PCA) was conducted to explore the patterns of association between soil physicochemical variables, water table depth and CH_4_ emission rates. The Kaiser–Meyer–Olkin's (KMO) measure of sampling adequacy was used to assess the usefulness of the PCA. KMO ranges from 0 to 1 and should be well above 0.5 if variables are sufficiently interdependent for the PCA to be useful [28]. The relationship between CH_4_ emission rates and measured rice field factors was analysed with Generalized Linear Models (GLMz). An information–theoretic approach was used to find the best approximating models [29]. GLMz were built including all possible combinations of independent variables, but excluding interactions, due to the large number of variables considered. Two additional criteria were used to define the candidate models: only those performing significantly better than the null model and those with a variance inflation factor of ≤ 5, in order to avoid multicollinearity effects in regression models [30]. The degree of support for each candidate model was assessed with the second order Akaike information criterion (AICc), rescaled to obtain *Δ*AICc values (*Δ*AICc = AICc_*i*_—minimum AICc). According to [29] (models having *Δ*AICc values within 1–2 of the best model have the most substantial support, those within *ca*. 4–7 have considerably less support, while models with *Δ*AICc > 10 have either essentially no support and might be omitted from further consideration. The relative plausibility of each candidate model was assessed by calculating Akaike’s weights (*w*_*i*_); *w*_*i*_ ranges from 0 to 1, and can be interpreted as the probability that a given model is the best model in the candidate set. Because no model was clearly the best one (*i*.*e*., *w*_*i*_ ≥ 0.9) model-average regression coefficients (*β*) were obtained as a weighted average (by model *w*_*i*_) of *β* across all models in which a given variable is present. The relative importance of each independent variable was calculated by the sum of *w*_*i*_ for all models in which a given variable occurs [29]. Finally, averaged *β* were compared with *β* from the full model to assess the impact of model selection bias on parameter estimates [31]. For all of candidate models, residuals showed to be normally distributed according to the Shapiro–Francia normality test (*P* > 0.10). Prior to the analysis, quantitative variables were transformed to improve linearity and homoscedasticity. Analyses were performed with the R software version 3.4.

## Results

### Environmental and agronomic traits in rice fields

**Fig 2:**
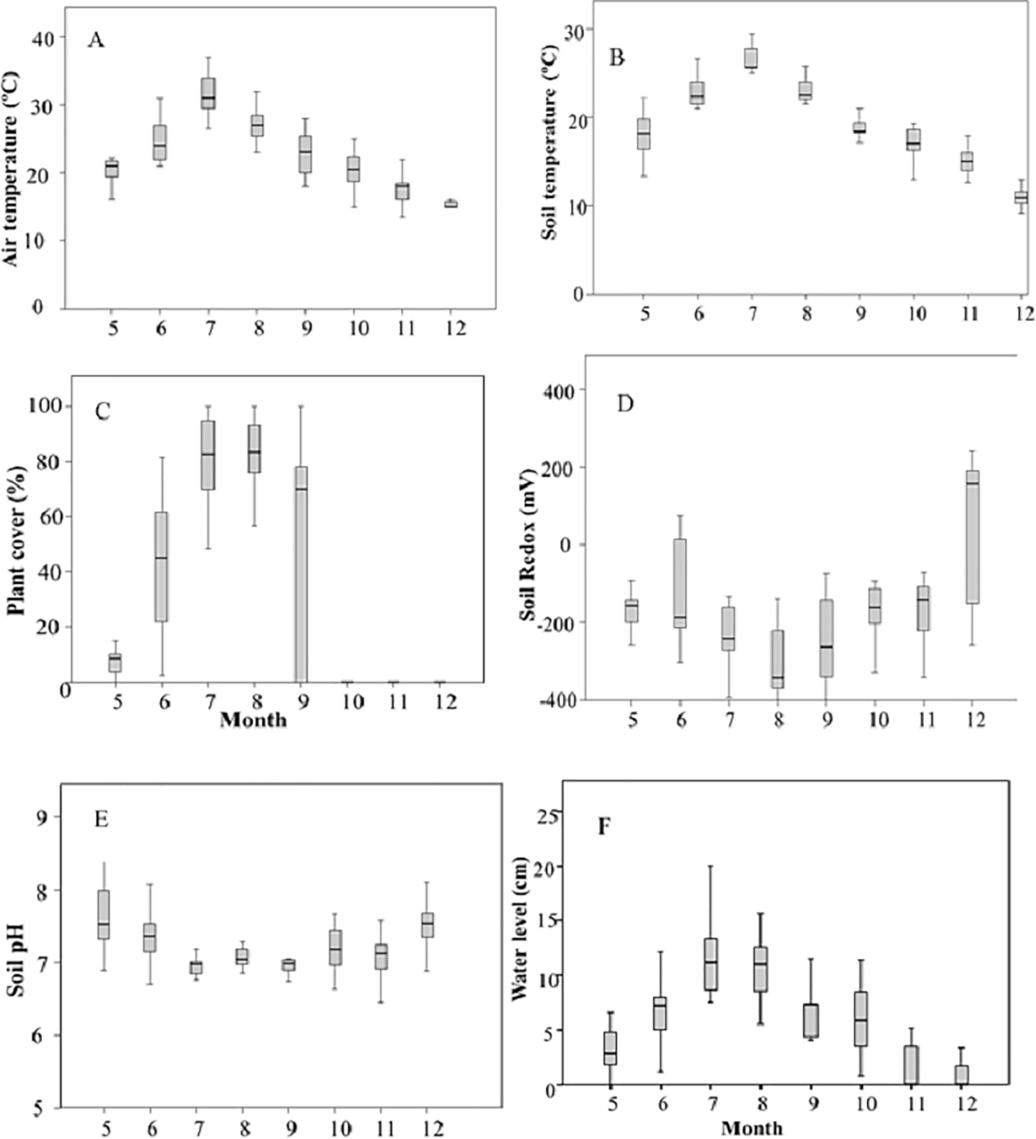
**Monthly seasonal variations of environmental variables (soil temperature, A; air temperature, B; soil pH, C; and soil redox, D) and agronomic (plant cover, E; water level, F) across the 15 monitored commercial fields**.

The 15 studied rice fields in the Ebre Delta were largely variable in soil properties (Table 1). The most common soil texture was silt loam (*N* = 6) followed by silty clay loam (4), silt loam (2), sandy clay loam (1), sandy loam (1) and loamy sand (1), thus showing a range from fine to coarse texture. Bulk density ranged from 0.688 to 1.104 g cm^-3^, organic matter from 1.89 to 4.94%, organic carbon from 1 to 2.8%, organic N from 0.11 to 1.82% and sulphates from 1266 to 5329 mg kg^-1^. The agronomic (water level and plant cover) and environmental (air and soil temperature, soil redox and soil pH) dynamics are shown in Fig 2. The fields were continuously flooded from the last week of April up to 7–10 days before harvest, in September. After direct wet seeding, which took place over the first two weeks of May, water levels were around 5–7 cm initially and gradually increased to around 15 cm (with increasing plant height) till mid-August, coinciding with the reproductive and grain filling stages of the crop. Thereafter, water irrigation was only supplied for a short period for straw incorporation operations which occurred either in October (in 9 out of the 15 fields) or November (4 rice fields) or December (2 rice fields) and then fields were progressively dried out. Agronomic variables, namely plant cover and water layer depth (hereafter, water level) followed the same pattern as soil and air temperature, *i*.*e*. progressive increase up to July/August, while the opposed pattern was observed for soil redox and soil pH. Negative soil redox (reductive conditions) were achieved in the first sampling, in May, approximately one month after the flooding of the fields (mean ± SE = – 153.3 ± 12.8 mV) and continued declining down to August (– 297.5 ± 14.7 mV). Thereafter, more variability across fields was found though a slight increasing tendency could be observed from September to December, when the mean soil redox was positive (64.8 ± 29.2 mV) because of the progressive drainage of the fields. Such variability resulted from different management practices (timing of harvest, timing of straw incorporation, water management) and soil textures that determined different drainage rates after the cut of the water supply. Soil pH turned from slightly alkaline in May (7.68 ± 0.14) to neutral in July (7.0 ± 0.03) until harvest (7.05 ± 0.07), and recovered the alkalinity condition at post-harvest (7.20 ± 0.40). The electrical conductivity remained quite constant over the growing (from July) and post-harvest seasons (1.1 ± 0.02 dS m^-1^).

**Table 1:** Soil traits characterizing the 15 commercial rice fields in the Ebre Delta.

Field #	Delta Bank	% Clay	% Silt	% Coarse silt	% Sand	Texture USDA	Bulk Density (g cm^-3^)	% Organic matter	% Total C	% Organic C	% N	Sulfates (mg kg^-1^)
**1**	South	24.1	53.9	12.4	9.6	sandy clay loam	0.969	3.42	6.61	2.6	0.21	2424
**2**	South	14.3	22.8	4.6	58.3	sandy loam	0.903	3.74	6.98	2.3	0.23	1928
**3**	South	34.6	51.5	7.6	6.3	silty clay loam	0.688	4.94	6.64	2.8	0.28	5329
**4**	South	23.1	50.7	16.9	9.3	silt loam	0.869	3.45	6.66	2.2	0.2	4417
**5**	South	29.3	47.6	9.5	13.6	silt loam	0.812	3.38	6.33	2.2	0.21	3688
**6**	North	29.6	52.3	10.5	7.6	silty clay loam	0.884	3.38	6.92	2.3	0.21	1983
**7**	North	28.5	35.7	7.8	28	silt loam	1.014	4.1	6.25	2.5	0.24	1357
**8**	North	14	21.1	4.1	60.8	sandy loam	1.016	2.95	6.41	1.8	0.18	1366
**9**	North	36.7	50.8	8	4.5	silt loam	0.878	4.12	6.50	2.7	0.25	3724
**10**	North	23.4	50.5	20.6	5.5	silt loam	0.879	3.05	6.40	2.3	0.19	1669
**11**	South	4.9	7.4	3.1	84.6	loamy sand	0.975	2.21	5.32	1.4	0.12	1266
**12**	North	25.1	38.1	20	16.8	silt loam	0.896	4.17	6.90	2.6	0.23	4394
**13**	South	35.3	51.6	6.9	6.2	silty clay loam	0.809	4.57	6.82	2.8	0.26	4433
**14**	North	21.3	26	4.5	48.2	loam	1.104	1.89	5.21	1	0.11	2244
**15**	South	9.8	11.6	3	75.6	loamy sand	1.013	3.54	5.77	2.2	0.19	4395

### Cumulative CH_4_ emissions and temporal pattern

Estimated cumulative CH_4_ emissions from May to December were 314.1 kg CH_4_ ha^-1^, and amounted a total of 6595.7 Tm yr^-1^ CH_4_ in the whole rice growing area (*ca*. 21125 ha). Estimated cumulative emissions were 98.4 kg CH_4_ ha^-1^ and 215.7 kg CH_4_ ha^-1^ during the growing season (May to September) and fallow season (October to December), respectively the later accounting for 68.7% of the total emitted CH_4_. Similarly, the average CH_4_ emission rate was near 4-fold higher in the post-harvest (9.8 ± 1.7 mg CH_4_ m^-2^ h^-1^) when compared to the growing season (2.7 ± 0.3 mg CH_4_ m^-2^ h-^1^). The mean annual emission rates were 5.2 ± 0.6 mg CH_4_ m^-2^ h^-1^.

**Fig 3:**
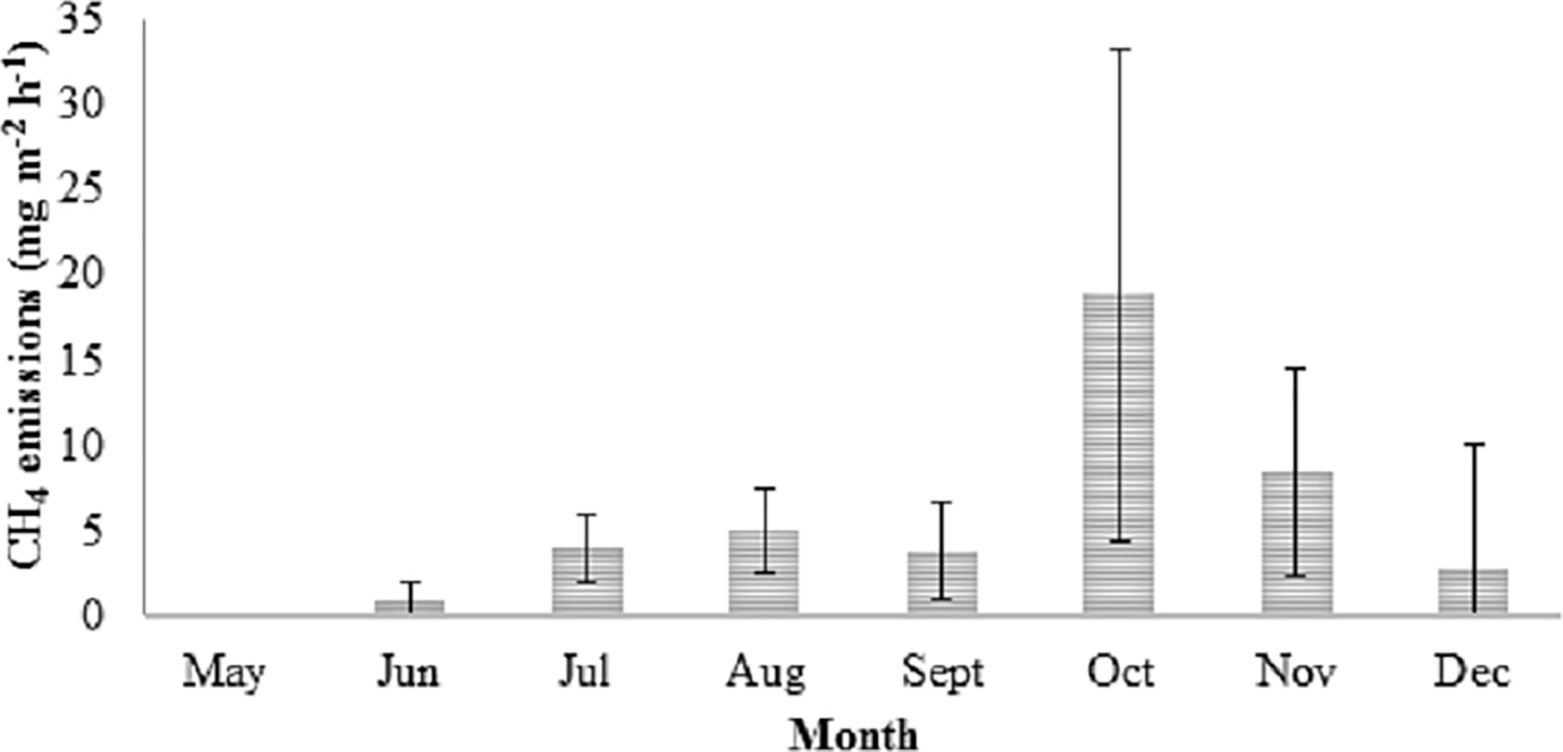
Monthly C-CH_4_ emissions rates in the Ebre Delta rice fields. Data presented are monthly averages (mg C-CH_4_ m^-2^ h^-1^ ± SE) across the 15 commercial rice fields. Annual, growing season and fallow season emission rates were 5.2 ± 0.62, 2.71 ± 0.25, and 9.71 ± 1.60 mg C-CH_4_ m^-2^ ha^-1^, respectively (in nmol m^-2^ s^-1^: 120.4 ± 14.34, 62.7 ± 5.8, 224.8 ± 37.0).

The onset of CH_4_ emissions occurred in June, several weeks after the inundation, with negligible emissions in May. The temporal pattern of the monthly mean emission rates (Fig 3) followed a bimodal distribution pattern with the first peak in August (5.0 ± 0.7 mg C-CH_4_ m^-2^ h^-1^) and the second one in October (20.2 ± 4.2 mg C-CH_4_ m^-2^ h^-1^). Temporal patterns of each monitored field can be found in S1 Fig.

### Agronomic and environmental drivers of CH_4_ emissions

**Fig 4:**
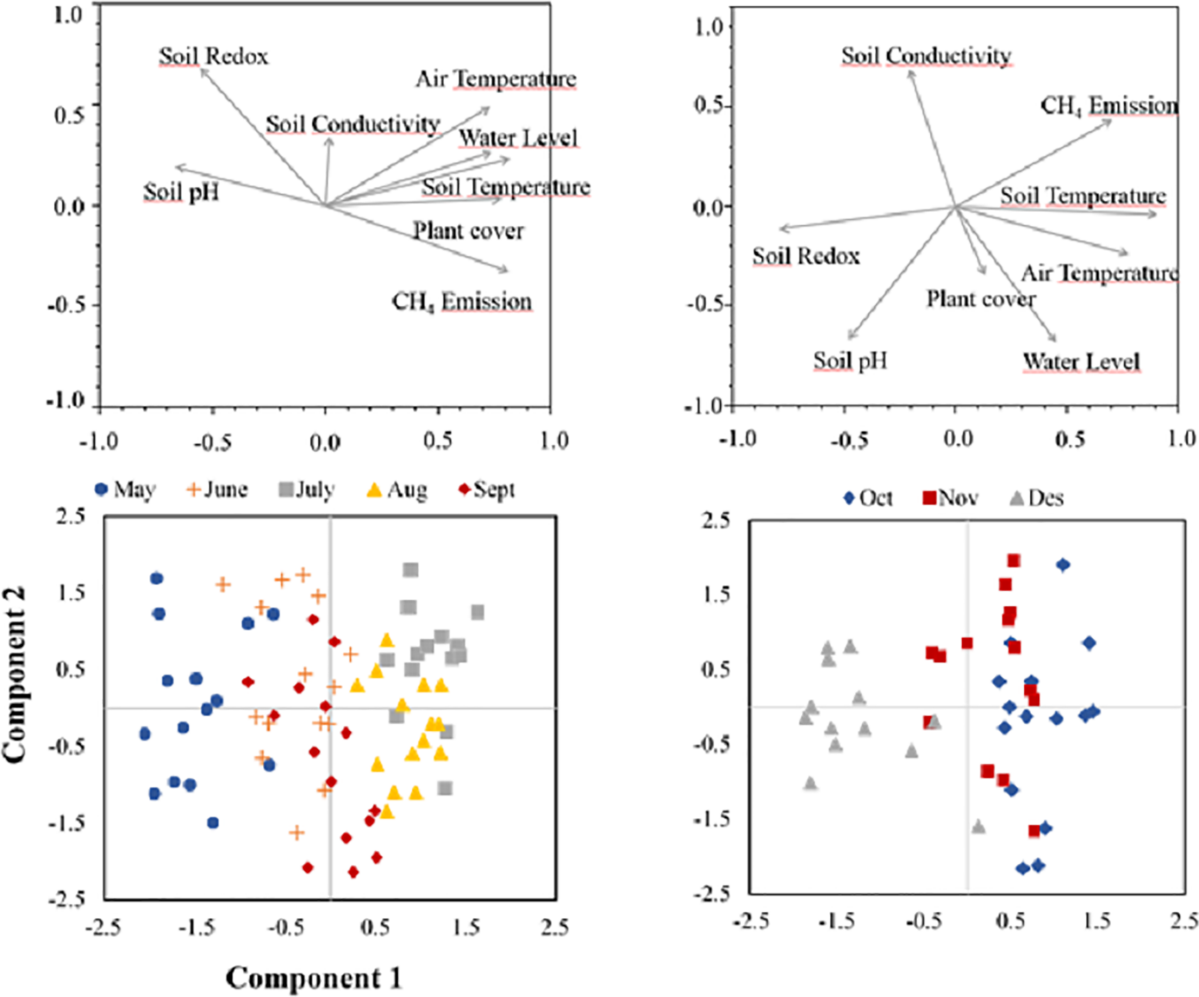
**Principal component analysis (PCA) of the monthly C-CH**_**4**_ **emission rates and soil physic-chemical variables for growing season (A) and off-season (B).** In 4 D, red correspond to October, green to November and blue to December; in E, light blue corresponds to May, purple to June, yellow to July, grey to August and black to September. Factor loadings of the variables and monthly scores on the first two principal component axes are shown.

**Fig 5:**
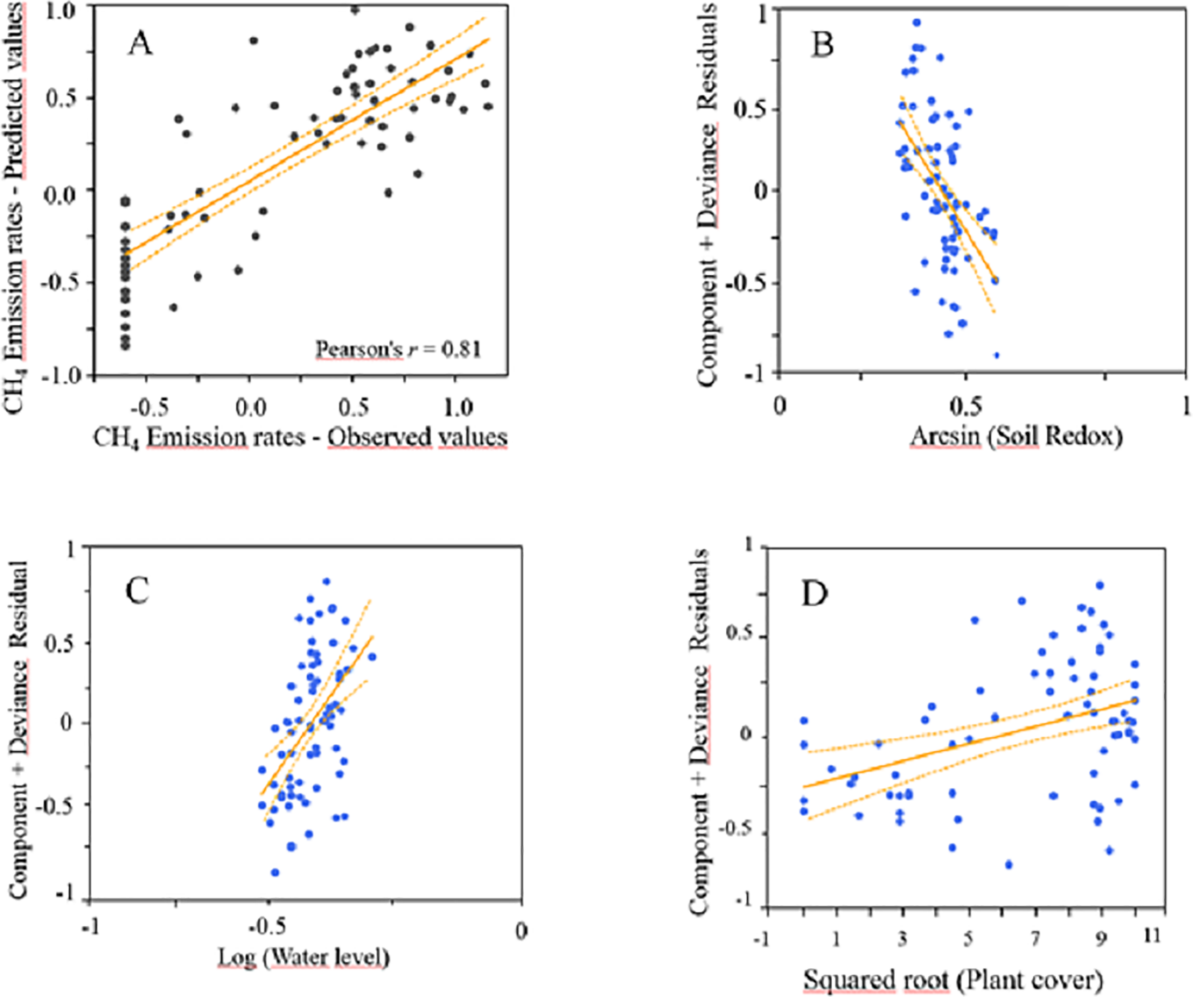
A) Relationship between the observed and the predicted C-CH_4_ emission rate values (mg C-CH_4_ m^-2^ h^-1^) over the in-season by the GLMz averaged through an information theoretic approach (see Table 2 for more details). B-D) Partial residual plots for the most influencing variables in C-CH_4_ emission rate. Partial residual plots show the effect of a given independent variable on the response variable given that all other independent variables are also included in the model. Solid lines shows the linear regression and the dashed lines are the 95% confidence interval for the regression line.

The PCA analysis conducted on rice field descriptors (both environmental and agronomic) and CH_4_ emission rates indicated that most of the analysed variables were interdependent and significantly intercorrelated. Rice harvest and different agronomic measures caused a sharp change in field physicochemical characteristics, clearly differentiating fallow and growing seasons, thus, different PCA were conducted independently (Fig 4). In the growing season, most of the analysed variables were also interdependent and significantly correlated between them. The KMO measure of sampling adequacy (0.80) indicated the usefulness of the PCA, with the first two explaining 47.5 and 14.0% of the total variation, respectively, over the rice growing season. The strongest correlations were found between soil temperature and air temperature (Pearson’s *r* = 0.69), between soil temperature and plant cover (0.60), and between CH_4_ and water level (*r* = 0.56), plant cover (*r* = 0.56), soil temperature (*r* = 0.51), soil redox (*r* = -0.57), and soil pH (*r* = -0.50). These latter, were opposed to both air and soil temperature, water level and plant cover (Fig 5). The first PCA axis summarised these correlations displaying a temporal pattern associated to the crop growth and the increasing temperature, and CH_4_ emissions over the summer. In contrast, the second PCA axis reflected field variations, mostly in soil redox, air temperature, conductivity and also CH_4_ emissions. (Fig 5). In the PCA (KMO = 0.68) for fallow season, the first PCA axis explained 40.5% of the total depicted variation (61.9%), summarizing the strongest correlations found; CH_4_ emissions, air temperature and soil temperature were positively correlated among them (*r* > 0.50), and opposed to soil redox (*r* > 0.55). The second PCA axis showed differences among fields, mainly related to water level and electrical conductivity, probably associated to different agronomic practices (Fig 4).

The information-theoretic framework analyses provided predictive models of the effects of the analysed variables on C-CH_4_ emission rates (Table 2). When season (*i*.*e*. growing and fallow) was included as factor, the AICc-based model selection selected 10 plausible models (*Δ*AICc < 7) to explain the variability in C-CH_4_ emission rates. The most influencing variables were season, soil redox, soil temperature (Selection Probability = 1), and soil pH (SP = 0.97), all of them included in the best AICc model. Soil conductivity, plant cover and water level were also selected, but had a weakest relationship with C-CH_4_ emissions (see SP values in Table 2). The analysis of the model averaged coefficients showed that soil temperature, plant cover and water level were directly related to C-CH_4_ emission rate, in contrast to pH and redox that were inversely related to C-CH_4_ emission rate. Season had also a negative relationship with C-CH_4_ emission rate, thus indicating lower emissions rates over the growing season.

**Table 2:** Results from the information-theoretic framework analysis to evaluate the variation of C-CH_4_ emission in the Ebre Delta rice field area.

model parameter	year model			in-season model			off-season model		
	N = 10			N = 20			N = 26		
	SP	ẞ	Bias	SP	ẞ	Bias	SP	ẞ	Bias
**(Intercept)**	1.000	4.125	-0.150	1.000	3.670	-0.191	1.000	-6.918	-0.115
**Soil Redox**	1.000	-4.579	0.0360	1.000	-3.798	0.026	0.453	-1.551	-1.142
**Soil Temperature**	1.000	3.253	-0.039	0.288	0.208	-2.977	1.000	4.771	-0.263
**Soil pH**	0.970	-5.975	-0.055	0.335	-0.766	-1.776	0.135	0.009	-218.89
**Soil conductivity**	0.324	-0.141	-2.200	0.379	-0.214	-1.400	0.230	0.221	-2.331
**Plant cover**	0.322	0.005	-2.503	0.956	0.050	0.021	0.240	0.021	-3.820
**Water level**	0.243	0.069	-0.479	1.000	3.884	0.103	0.985	-5.240	0.044
**Air temperature**		NS		0.225	0.000	1721.8	0.203	-0.360	-3.823
**Season**	1.000	-1.233	-0.028						
**Straw 1**		NI					0.993	0.788	-0.156
**Straw 2**		NI					0.993	-0.001	2.457
**Straw 3**		NI					0.993	-0.556	0.703

Model-averaged regression coefficients (ẞ) are parameter coefficients averaged by model weight (w_i_) across all candidate models (ΔAICc < 7) in which the given parameter occurs; selection probability (SP) indicates the importance of an independent variable, and parameter bias is the difference between the averaged estimates (ẞ) and the full model coefficients. The number (N) of candidate models (ΔAICc < 7) is also shown. Parameters included in the best model, in each case, are highlighted in blue colour. Abbreviation: NS, Not selected by the model; NI, not included.

**Fig 6:**
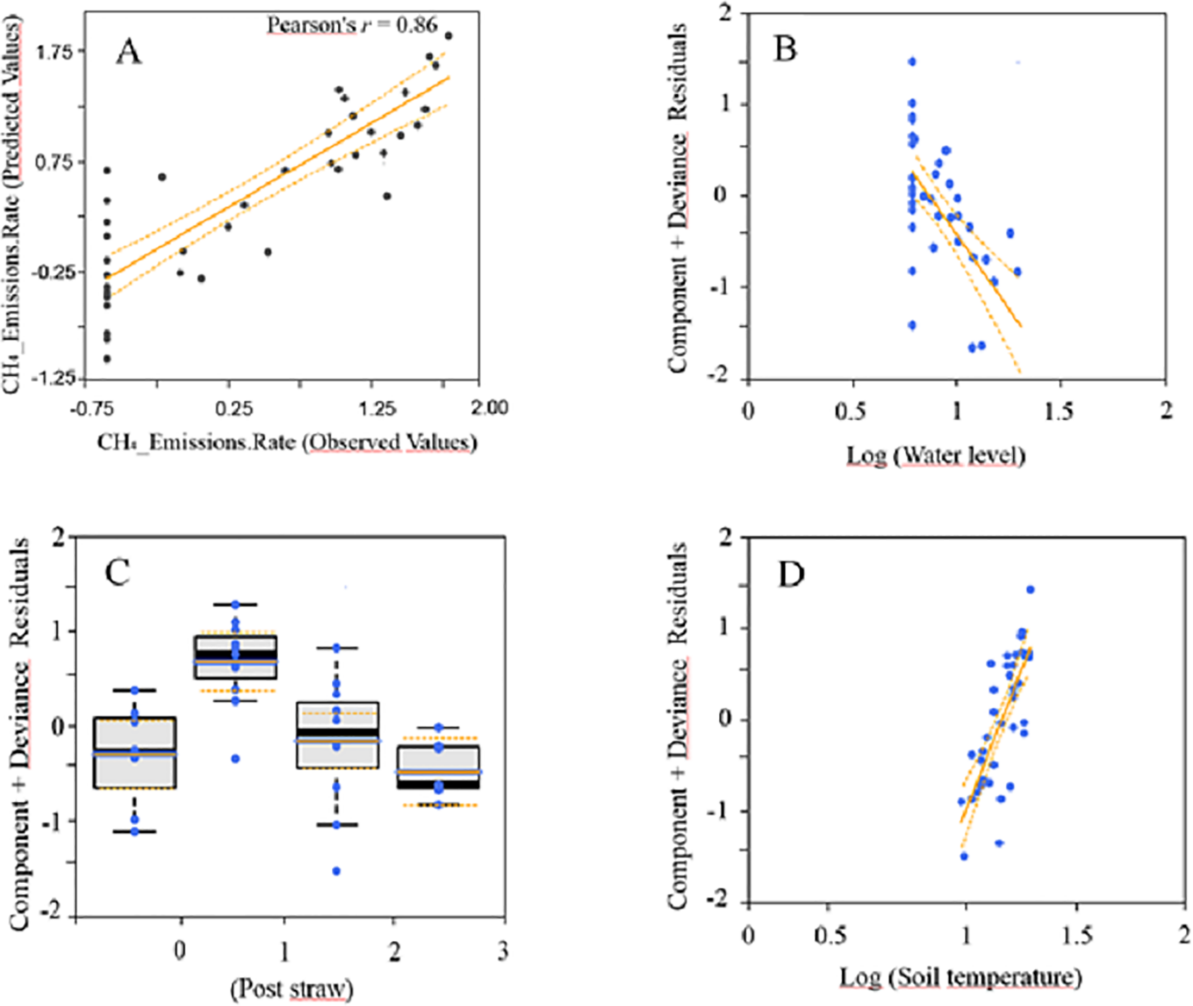
A) Relationship between the observed and the predicted C-CH_4_ emission rate values (mg C-CH_4_ m^-2^ h^-1^) over the off-season by the GLMz averaged through an information theoretic approach (see Table 2 for more details). B-D) Partial residual plots for the most influencing variables in CH_4_ emission rate. Partial residual plots show the effect of a given independent variable on the response variable given that all other independent variables are also included in the model. Solid lines show the linear regression and the dashed lines are the 95% confidence interval for the regression line.

When both seasons were analysed separately (in-season and off-season models in Table 2), the correlation between predicted and observed values was significant, (Pearson's *r* = 0.81, *P* < 0.0001; and *r* = 0.85, *P* < 0.0001; for growing and fallow seasons, respectively), supporting the predictive ability of the models (Table 2, Figs 5 and 6), but contrasting patterns were found. While water level and plant cover (positively related), and soil redox (negatively related) were the most important variables in explaining differences in CH_4_ emissions rates over the growing season, in the fallow season the most important variables were soil temperature (directly related), water level (inversely related), and straw incorporation (see SP values in Table 2). Straw refers to the timing of rice straw incorporation, thus Straw 1, Straw 2 and Straw 3 indicates the two months after straw incorporation. Consequently, CH_4_ emissions rate was highest immediately after the rice straw incorporation, and declined over the following months (Fig 6), with Straw 2 showing similar C-CH_4_ emissions to Straw 0 (the sampling previous to rice straw addition). Water level was one of the most important variables over both growing and fallow seasons, but, interestingly, had opposite effects on CH_4_ emissions between seasons (Table 2, Figs 5 and 6). Among the remaining variables, soil conductivity, soil pH and air temperature were less influencing variables in explaining CH_4_ emission rate differences, and also showed different effects between seasons (see SP and *β* values in Table 2). Soil temperature and plant cover had also some minor effects in both in-season and off-season respectively.

## Discussion

### Cumulative CH_4_ emissions: Temporal pattern and spatial heterogeneity

Cumulative estimates on annual CH_4_ emissions from the Ebre Delta rice fields amounted 314 kg CH_4_ kg ha^-1^, of which 68.7% (215.7 kg C-CH_4_ ha^-1^) were emitted during post-harvest. In comparison with other Mediterranean and temperate rice growing areas, the cumulative emissions during the growing season (98.4 kg CH_4_ ha^-1^) are in good agreement with those previously reported in Spain [7] and Portugal [8] but are in the lower range of those in the USA, which ranged 85–338 kg CH_4_ kg ha^-1^ [24, 32–36] and lower than those reported Japan by [10, 37, 38] which ranged from 121.4 to 291.3 kg CH_4_ ha^-1^. The observed values are also remarkably lower than those reported in Italy, between 370 and 413 kg CH_4_ kg ha^-1^ [16, 21, 39]. Studies on CH_4_ emissions have been conducted extensively since the 1990s in monsoon Asian countries since 90% of the world’s rice fields are located in Asia, as compiled by [40]. Because of the large differences between Asian and Mediterranean and temperate rice growing systems (climate, number of rice crop per year, planting system, cultivars), emission estimates are expected to differ significantly, so they cannot be extrapolated from one region to another. Hence, the present paper is centred in a Mediterranean climate context in order provide a more agroclimatic-focused and specific understanding of CH_4_ emission dynamics.

The herein presented CH_4_ emissions from the Ebre Delta rice fields are derived from a multi-site experimental design as opposed to most of the studies conducted so far in the Mediterranean and temperate climate regions which base their estimation on single-site studies. Multi-site studies improve the accuracy of the estimates because allow covering most of the spatial agronomic and environmental heterogeneity as seen in the variability of the measured variables (Fig 2). Such variability has been studied through multivariate analyses in order to assess the relative contribution of each variable to CH_4_ emission. However, in our case its implementation was at the expense of sampling frequency so that temporal variability could be to some extent underestimated. We are aware that for some critical stages we could have missed some variations in the CH_4_ emissions, *i*.*e*. during the application of fertilizer, when draining the fields for herbicide spraying, etc. Covering both spatial and temporal heterogeneity is important to improve the accuracy of the estimates and efforts should be made in increasing sampling frequency in multi-site experiments.

Likewise many studies on GHG emissions in rice fields [8, 16, 21, 24, 36], we used lineal regression to estimate CH_4_ emission rates, as opposed to other authors advocating non-linear models [41–44]. Sampling procedures associated to chamber deployment such as soil disturbance [45], duration of sampling and chamber size [44, 46] may induce departure from linearity. However, the significance of the linear functions, the low soil disturbance we caused thanks to the floatability and non-insertion of our chambers into the soil and the individual observation of gas exchange pattern in chambers allow us to relay on the linearity of the adjustment.

The present study is also the first attempt in measuring CH_4_ emissions during the fallow season in Spain and the observed large contribution of this stage to the annual CH_4_ emissions points out two important facts; first, it is necessary to take into account the fallow season to improve the accuracy of the estimations and; second, there is a need of further studies concerning mitigation practices during the fallow because of the expected higher impact on emission.

Global and national inventories of greenhouse gases are necessary to estimate spatiotemporal variability and trends of emissions at different geographical and sectorial scales. IPCC provides tiered methodology for estimating and reporting CH_4_ emissions for rice cultivation [47]. Tier 1 methodology provides default emission and scaling factors for those regions lacking specific emission factors, as is the case of Spain, and it only considers the growing season. Accordingly, we applied Tier 1 methodology to the Ebre Delta rice fields, and the result was 8.17 mg CH_4_ m^-2^ h^-1^ and 230.4 kg ha^-1^ of total CH_4_. The comparison between estimated (IPCC, Tier 1) vs observed emissions (present study) shows that growing-season emissions are overestimated by 57% but because such methodology does not consider off-season emission, the annual emissions are underestimated by 41%. Such underestimation is likely to occur in regions where rice is cultivated as a mono-crop with similar management in the fallow or inter-cropping seasons, as it is the case of California [24], Portugal [48] and Italy [21].

### Agronomic and environmental drivers of CH_4_ emission rates in the growing season

The agronomic (flooding regime, water layer depth, plant cover and timing of straw incorporation) and environmental (soil redox, temperature, soil pH and conductivity) variables were highly correlated between them and in relation to CH_4_ emissions. The inundation of the fields reduces soil redox over the flooding period, turns soil pH from alkaline to neutral [49, 50] and reduces soil electrical conductivity. The positive correlation of water level to temperature (soil and air) and plant cover is driven by the implemented water management since farmers increase water level with the advance of the summer to prevent heat and salinity stress to the crop.

Our study reveals that, during the growing season, soil redox, water level and plant cover are the main drivers of CH_4_ emission rates. Soil redox is one of the most critical factors for CH_4_ emission. The production of CH_4_ is initiated within a– 100 to –210 mV range of soil redox potential which is achieved several weeks after the flooding of rice fields, between May and June. Thereafter, CH_4_ emissions proportionally increase as the soil become more reductive following a sequential depletion of alternative terminal electron acceptors–such as NO_3_^-^, Fe^3+^, Mn^4+^ and SO_4_^2-^, prior to acetate/H_2_ oxidation and CO_2_ reduction to generate CH_4_ during methanogenesis [51, 52].

Emission of CH_4_ started in June, increased with rice growth, reached a maximum at plant cover around 80% (July or August) and declined thereafter, in September. This pattern shows the linkage between CH_4_ production and photosynthesis through the production of plant biomass [53] and the consequent proportional availability of soil labile carbon through excretion of root exudates [54] and deposition of plant residues, providing organic substrates for methanogens [55]. In addition, the vegetative development is proportional to the CH_4_ transport capacity from the soil to the atmosphere through the aerenchyma [56]. The drop in methane emissions at maximum plant cover correspond to fields at an early ripening stage, which have a lower transport capacity because of leaf senescence [57] and reduced carbon supply from plant photosynthates at the end of the growing season [58].

We did not find a particular phenologic stage associated to CH_4_ peaks as maximum rates were observed from late reproductive stage (late July), early grain filling stages (August) and even ripening (September). This variability is in line with the contrasting stages associated to CH_4_ peaks reported in the literature: tillering [59, 60], panicle initiation [61], heading [55] and maturity or [23, 62]. This heterogeneity is presumably explained by the presence of other factors modulating the seasonal pattern of emissions. For instance, another source of variation could be the concentration of sulfates in the soil before sowing, which ranged from 1266 to 5329 mg kg^-1^ across the fifteen fields (Table 1), because in the presence of sulfates, sulfate-reducing bacteria outcompete the methanogens for hydrogen and acetate utilization [63].

Water table acts as an “*on-off switch*” as already described by [64]but there is also a positive response of CH_4_ emissions to the increase of water level, likely because of an indirect positive effect on the soil anoxic layer [65] resulting in larger volume of soil with appropriate conditions for methanogenesis. However, it is worthy to note a possible collinear effect of water level with the crop development, as water level is progressively increased by farmers.

### Agronomic and environmental drivers of CH_4_ emission rates in the fallow season

In the fallow season, the major drivers of CH_4_ emission were soil temperature, water level and straw incorporation, which differ from those in the growing season or show different associations with CH_4_ emission rates, as in the case of water level.

After harvest, fields were left to naturally drain and irrigation was only supplied during the operation of straw incorporation. Overall, the peak of CH_4_ emissions in the fallow occurred in October, but looking at the fields individually, and according to the GLMz analyses, the maximum CH_4_ emission rate in the fallow occurs a short period after straw incorporation, no later than one month after straw input, which happened in October in 9 fields, in November in 4 fields and in December in 2 fields. The monthly frequency of gas sampling does not allow us to be precise in the time gap between straw incorporation and the peak of emissions which could be explained by two processes. The first would be a rapid response of the methanogenic community to the large straw input [66, 67]. The incorporation of a large amount of organic matter could have stimulated microbial hydrolytic activities under oxic/anoxic conditions resulting in the accumulation of fermentation products hence boosting methanogenesis in the anoxic layer. The second, an escape of the CH_4_ entrapped into the soil which would have been produced and accumulated over the growing season [68] and eventually released because of soil disturbance caused by the mechanical operations during straw incorporation.

Elucidating which is the major route of CH_4_ emissions has important implications in designing mitigation strategies. If the major emission mechanism is linked to fallow-season methanogenesis, mitigation practices during the fallow have potentially stronger effect than those applied during the growing season. Conversely, if methane entrapment is more significant (CH_4_ released because of soil disturbances), that would balance the relative contribution of fallow over growing emissions and so mitigation strategies to be implemented during the two periods would be equally important.

Our study does not address the fraction of CH_4_ evolved from each of these two above described processes and the low sampling frequency impedes the determination of their relative contribution to the emission patterns. In case soil entrapment is the dominant route, the probability of capturing such CH_4_ pulsed emissions during our monthly sampling is low. The dominant effect of soil temperature on fallow season CH_4_ emission rates (later discussed) suggests that fallow methanogenesis might be preponderant. Besides, CH_4_ entrapped into the soil throughout the growing season is reported to be released after the drainage prior to harvest [22, 23, 69] and, thus, it should have been released before the harvest. In contrast, the entrapment route could be supported by the low contribution of soil redox to CH_4_ emission, suggesting that the methanogenic activity would not be proportional to the CH_4_ emission rate, although it is also true that such lack of proportionality could, in turn be explained by a stronger effect of large substrate availability in relation to kinetic microbial [70, 71]. Hence, additional research based on a more intense sampling around straw soil incorporation should be conducted to determine the dynamics of CH_4_ emissions to eventually be able to give a deeper insight into the underlying processes explaining off-season CH_4_ emissions.

While the moment of straw incorporation determines the pulse of CH_4_ emissions in the fallow season, soil temperature modulates the magnitude of this peak: when the straw was incorporated in October, soil temperature and mean emission rates were higher (17.2 ± 1.8°C, 28.7 ± 7.2 mg CH_4_ m^-2^ h^-1^) than those recorded in November (15.5 ± 1.7°C, 6.1 ± 3.2 mg CH_4_ m^-2^ h^-1^) and in December (11.0 ± 1.2°C, 19.3 ± 17.7 mg CH_4_ m^-2^ h^-1^). The effect of temperature on CH_4_ emission have been reported before [72] and in particular during the winter season [73]. It is worthy to note the high mean emission rates observed in one of the two fields in which straw was incorporated in December (P15) with 37.0 mg CH4 m^-2^ h^-1^ compared to the other plot (P11) with 1.5 mg CH_4_ m^-2^ h^-1^. The time gap between straw incorporation and CH_4_ sampling could be another of the reasons, so that the closer the straw input to gas sampling the higher the CH_4_ peak [72] but we don’t have this data. Thus, at this stage we are not able to reliably suggest a reason to explain the high emissions rates recorded in December.

A number of studies have been conducted on the effect of straw input on CH_4_ emissions in the following growing season [74–76]. Using meta-analysis, [77] concluded that straw return to rice paddies increases by 117% the CH_4_ emission during the following rice growing season and [78] found that such increase is proportional to the total amount of added straw.

Studies on CH_4_ emissions during the fallow or inter- conclude that lower CH_4_ is emitted either when straw is removed or when it is incorporated but fields are drained thereafter [16, 24, 79, 80]. In contrast, substantially higher CH_4_ emissions are reported with straw input plus winter flooding, accounting from 23% to 61% of annual emissions [24, 34, 81] and even 70%, as shown in the present study. According to this, straw should be removed from the fields to mitigate CH_4_ emissions or fields should be drained after straw input. However, straw return has been proven to show agronomic and enviromental benefits such as increases in grain yield [82], improvement of soil fertility [83] and to enhance soil C sequestration [84] which could offset, at least partially, the net negative impact of CH_4_ emission. In regard to the field drainage after straw input option, it should be taken into account that draining the fields after the incorporation of the straw may result in either high CO_2_ emissions under aerated of soil [85] or promote early CH_4_ emissions in the following growing season [86, 87] that could compensate or even increase the overall warming potental. Mitigation strategies for straw management should be identified to keep the benefits of straw return while minimizing fallow CH4 emissions: for instance, the removed straw could be used to generate biogas in external anaerobic digestors [88, 89] and the resulting digestates could still have fertiliazing value when returned to the soil [90]. In addition, the role of temperature modulating CH_4_ emissions during and immediately after the straw incorporation suggests that delaying straw input within the fallow season could also be considered as a mitigation practice.

### Different drivers of CH_4_ emission rates in the growing and post-harvest seasons

Our study shows that the main factors controlling CH_4_ emissions differ between the growing and fallow seasons. Soil temperature is a limiting factor for CH_4_ emissions during the fallow whereas it becomes less relevant during the growing season. The relatively low temperatures recorded during the fallow season (9 − 21°C) compared to the optimum (35 to 42°C) for methanogenesis [91, 92] could have limited methanogenic activity, whereas less influence of temperature was detected during the growing season, were soil temperature ranged from 13–30°C but was mostly above 20°C. On the other hand, variations in agricultural practices and their timings across the rice fields during the cultivation period could have become more relevant than the relationship between soil temperature and CH_4_ emissions, as opposed to the fallow when a simpler and more homogeneous management is conducted. The effects of temperature on CH_4_ emissions in paddy rice during the growing season are controversial: while some studies report weak or no correlation between methanogenesis and CH_4_ emissions during the growing season [8, 23, 62, 93] others report a positive correlation [7, 13, 55]. The lack of an integrative approach of the multi-factor effect of agronomic and environmental variables on CH_4_ emissions could have brought about such inconsistency across studies.

The weaker contribution of soil redox to CH_4_ variability during the fallow contrasts to the relevance of this factor during the growing season: it may suggest a lower contribution of the methanogenic activity or it might be given by the stronger effect of large substrate availability (straw input) relative to methanogen kinetics. On the other hand, such apparent contradiction might be also explained by differing rates of redox reactions of microbial communities. Opposed trends were also observed for the relationship between CH_4_ emission rates and water layer depth. Growing CH_4_ emission rates in the growing season increased with water depth whereas the negative contribution during the fallow may suggest either methanotrophy in the water column, an effect of temperature on CH_4_ solubility in water [94] or simply a collinear effect to the straw input operations.

## Conclusions

The novelty of our investigation is based on the farm-to-farm CH_4_ emissions variation that allowed a multivariate analyses involving agronomic and environmental variables and their influence in CH_4_ emissions.

The estimated annual cumulative CH_4_ emissions in the Ebre Delta rice fields were 314.1 kg CH_4_ ha^-1^ of ca. 70% were emitted in the fallow season. The temporal pattern followed a bi-modal distribution with two peaks, the first in August and the second in October.

Differing controlling factors to CH_4_ emission in the growing (soil redox, water level and plant cover) and fallow (soil temperature, straw incorporation and water level) were identified. The emission rates during the growing season are closely associated to the rice crop development: CH_4_ emissions increase with plant cover and with the duration of the flooded conditions (since redox becomes more reductive) and with water level, which is progressively increased by farmers with the plant growth. In the fallow season, CH_4_ emissions are largely determined by the incorporation of rice straw into the soil. The relative contribution of the underlying mechanisms explaining the large fallow CH_4_ emissions could not be determined in this study because of low sampling frequency, though several options are discussed in the paper.

It is derived from this study that straw and water management during the fallow season may be fundamental to determine effective mitigation strategies, finding that could prompt and orientate new research in field. Ideally, new studies should be based on multi-site experiments and on intensive sampling frequency while the whole global warming potential assessed, thus including CH_4_, N_2_O and CO_2_ emissions.

## Supporting information

S1 Fig Monthly emission rates of C-CH4 (average, mg m^-2^ h^-1^, ± SE) in each monitored commercial rice field (P1 –P15) in the growing season, from May to September (Top), and in the fallow season, from October to December (Bellow). (TIF)

S1 Text Kellogg’s-LIFE agreement for sampling rice fields. (PDF)

S1 Table 2015_CH4 Ebre Delta Raw data. (XLSX)
