# Correction: Neglecting the fallow season can significantly underestimate annual methane emissions in Mediterranean rice fields

**DOI:** 10.1371/journal.pone.0202159

**Published:** 2018-08-08

**Authors:** Maite Martínez-Eixarch, Carles Alcaraz, Marc Viñas, Joan Noguerol, Xavier Aranda, Francesc Xavier Prenafeta-Boldú, Jesús Antonio Saldaña-De la Vega, Maria del Mar Català, Carles Ibáñez

## Notice of Republication

An incorrect version of S1 Table was published in error. This article was republished on July 25, 2018 to correct for this error. Please download this article again to view the correct version.
