# Supplementary figures and images for: Neglecting the fallow season can significantly underestimate annual methane emissions in Mediterranean rice fields
Source: PLoS One. 2018 May 31;13(5):e0198081. doi: 10.1371/journal.pone.0198081 (PMC5978985; doi:10.1371/journal.pone.0198081)

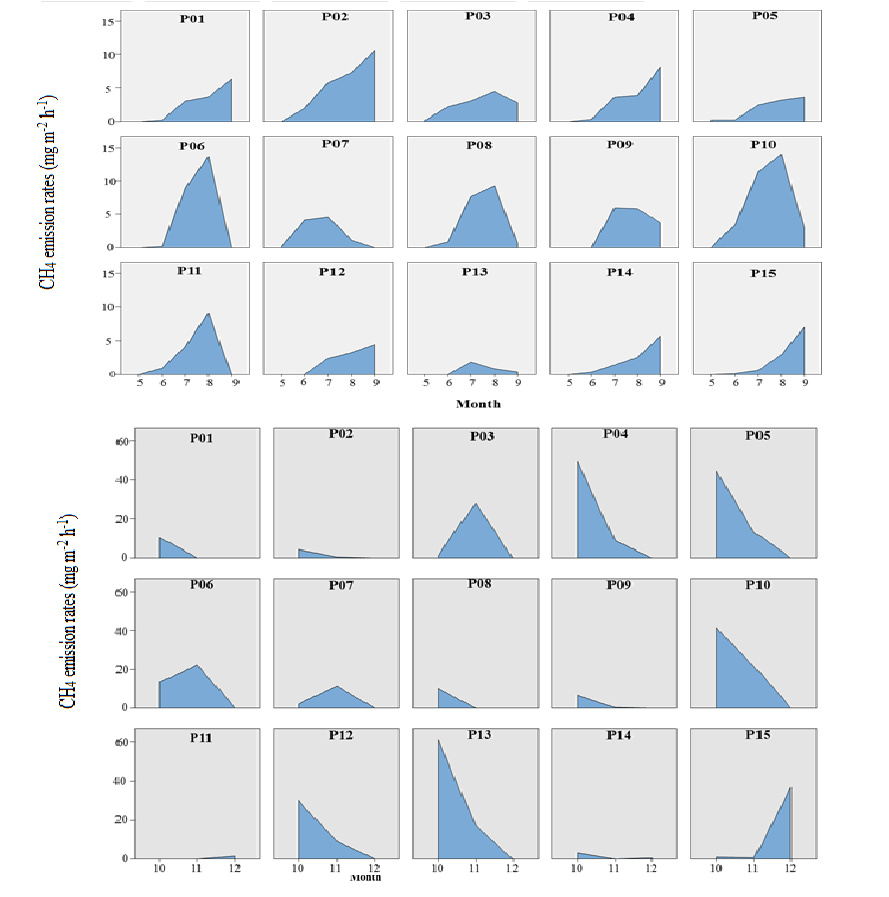

Supplement: S1 Fig — (TIF) [file pone.0198081.s001.tif]
