# Supplementary material for: Neglecting the fallow season can significantly underestimate annual methane emissions in Mediterranean rice fields
Source: PLoS One. 2018 May 31;13(5):e0198081. doi: 10.1371/journal.pone.0198081 (PMC5978985; doi:10.1371/journal.pone.0198081)
Supplement: S1 Text — (PDF) [file pone.0198081.s002.pdf]

Acuerdo de colaboración entre el Proyecto LIFE+ EBRO-ADMICLIM del IRTA y el Programa ORÍGENES de KELLOGG'S

El proyecto EBRO-ADMICLIM, cofinanciado por el Programa LIFE+ de la Unión Europea, es un proyecto piloto de medidas de mitigación y adaptación al cambio climático en el Delta del Ebro. Está liderado por el IRTA y en calidad de socios participan instituciones como la *Oficina Catalana del Canvi Climàtic* de la *Generalitat de Catalunya*, la *Agència Catalana de l'Aigua*, la *Comunitat de Regants-Sindicat Agrícola de l'Ebre*, el *Consorci d'Aigües de Tarragona*, el *Institut Cartogràfic i Geològic de Catalunya* y la *Universidad de Córdoba*. Una de las acciones del proyecto consiste en la optimización de las emisiones de gases de efecto invernadero (GEI) en los arrozales del Delta del Ebro. Para este propósito, se pretende monitorizar la emisión de GEI en campos de arroz representativos del cultivo en el Delta del Ebro y establecer relaciones con las prácticas agrarias. El Dr. Josep Carles Ibáñez es el Director del proyecto EBRO-ADMICLIM.

La empresa Kellogg's ha lanzado el programa *Orígenes* con el objetivo de promover la sostenibilidad del cultivo del arroz en el Delta del Ebro a través del seguimiento del cultivo y la aplicación de buenas prácticas agrícolas. El programa se realiza bajo la supervisión y asesoramiento del IRTA en campos de arroz de agricultores integrantes de este programa. El Sr. Richard Burkinshaw, director del programa de sostenibilidad de Kellogg's en Europa, es el responsable del programa *Orígenes* en el Delta del Ebro.

De acuerdo con la voluntad de ambas partes de mejorar la sostenibilidad del cultivo del arroz en el Delta del Ebro y contribuir así a la adaptación y mitigación del cultivo a los efectos del cambio climático, mediante la presente carta se establece un acuerdo de colaboración entre el Proyecto LIFE+ EBRO-ADMICLIM y el programa ORÍGENES de Kellogg's. Esta colaboración consiste en facilitar al equipo técnico del proyecto EBRO-ADMICLIM la autorización de los propietarios para realizar valoraciones (muestreos para análisis de GEI, agua y suelos) en los campos de arroz pertenecientes al Programa Orígenes, así como tener acceso al registro de las prácticas agrarias de los agricultores. Por su parte, el IRTA se compromete a transferir los resultados obtenidos a los integrantes del programa Orígenes de Kellogg's.

Y para que así conste a los efectos oportunos, firman el presente acuerdo de colaboración

Sr. Richard Burkinshaw
Director del programa de sostenibilidad
Kellogg's

Dr. Josep Carles Ibáñez Martí
Director del proyecto LIFE-EBROADMICLIM
IRTA

En St. Carles de la Ràpita, a 9 de Enero de 2015
